# Supplementary material for: Addressing Malnutrition Through Reducing the Cost of a Healthy Diet in Bangladesh
Source: Foods. 2025 Dec 10;14(24):4237. doi: 10.3390/foods14244237 (PMC12731697; doi:10.3390/foods14244237)
Supplement: Supplementary file 1 [file foods-14-04237-s001.zip › foods-4001388-supplementary.pdf]

Supplementary Table S1. List of participants presented in the expert consultation workshop.

**Organization**	**Name**	**Designation and Organization**
Ministry of Food (MoFood)	Md Masudul Hasan	Secretary, Ministry of Food
Food Planning and Monitoring Unit (FPMU)	Md Mahbubur Rahman	Director General, FPMU
Food Planning and Monitoring Unit (FPMU)	Feroz Al Mahmud	Research Director, FPMU
Food Planning and Monitoring Unit (FPMU)	Mostafa Faruq Al Banna	Research Director, FPMU
Food Planning and Monitoring Unit (FPMU)	Mohammad Abul Hashem	Research Director, FPMU
Food Planning and Monitoring Unit (FPMU)	Mezanur Rahaman	Associate Research Director, FPMU
Food Planning and Monitoring Unit (FPMU)	Md. Mahinur Islam	Associate Research Director, FPMU
Food Planning and Monitoring Unit (FPMU)	Alima Nushrat Jahan	Associate Research Director, FPMU
Food Planning and Monitoring Unit (FPMU)	Mohammad Ismail Mia	Associate Research Director, FPMU
International Fund for Agricultural Development (IFAD)	Valantine Achancho	Country Representative, IFAD
International Fund for Agricultural Development (IFAD)	Mashiat Chowdhury	Country Programme Analyst, IFAD
World Bank (WB)	Amadou Ba	Senior Agricultural Economist, World Bank
World Food Programme (WFP)	Domenico Scalpelli	Country Representative, WFP
World Food Programme (WFP)	Riccardo Suppo	Global Network Program Officer, WFP
World Food Programme (WFP)	Dohyeon Mun	Programme Policy Officer, WFP
Food and Agriculture Organization of the United Nations (FAO)	Jiaoqun Shi	Country Representative, FAO
Food and Agriculture Organization of the United Nations (FAO)	Dia Sanou	Deputy Country Representative, FAO
Food and Agriculture Organization of the United Nations (FAO)	Dr. Sreekanta Sheel	FAO
World Health Organization (WHO)	Faria Shobnom	WHO
United Nations Children's Fund (UNICEF)	Deepika Sharma	UNICEF
UNICEF	Golam Mohiuddin Khan Sadi	UNICEF
International Food Policy Research Institute IFPRI	Md. Jainal Abedin	Country Coordinator, SHIFT, IFPRI
Foresight for Food Systems Transformation (FosTr) team	Dr. Jim Woodhill	Lead, Foresight4Food Initiative
Foresight for Food Systems Transformation (FosTr) team	Just Dengerink	Country Facilitator, Wageningen University & Research
Foresight for Food Systems Transformation (FosTr) team	Sophie Galema	Reseracher Food Systems, Wageningen University
Shamba Centre For Food and Climate	Oshani Perera	Co-Founder,Shamba Centre for Food & Climate
Global Alliance for Improved Nutrition (GAIN)	Andoni Santamaria Kampfner	Senior associate, KL, GAIN
Global Alliance for Improved Nutrition (GAIN)	Zakir Hossain Akanda	Senior Advisior, GAIN
Global Alliance for Improved Nutrition (GAIN)	Sanjay Kumar Bhowmik	Senior Advisior, GAIN
Global Alliance for Improved Nutrition (GAIN)	Subir Bhadra	HOO, GAIN
Global Alliance for Improved Nutrition (GAIN)	Dr. Rudaba Khondker	Country Director, GAIN
Global Alliance for Improved Nutrition (GAIN)	Dr. Mohammad Monirul Hasan	Country Adviser, GAIN
Global Alliance for Improved Nutrition (GAIN)	Dipanjan Adhikary Protyai	Program Assistant, GAIN
Global Alliance for Improved Nutrition (GAIN)	Nazia Ahmed	Consultant, GAIN
Global Alliance for Improved Nutrition (GAIN)	Ruhul Amin Talukder	Senior Advisior, GAIN
Global Alliance for Improved Nutrition (GAIN)	Khondakar Mostan Hossain	Senior Advisior, GAIN
Global Alliance for Improved Nutrition (GAIN)	Shahnaz Arefin	Senior Advisior, GAIN
Global Alliance for Improved Nutrition (GAIN)	Gazi Mohammad Najmus Sakib	GAIN

Organization	Name	Designation and Organization
Global Alliance for Improved Nutrition (GAIN)	Esfar Ahmed Adittya	Consultant, GAIN
Directorate General of Food	Md Abul Hasanath Humayun Kabir	Director General, Directorate General of Food
Ministry of Agriculture (MoA)	Dr. Md. Mahmudur Rahman	Additional Secretary, PPC wing MoA
Ministry of Fisheries and Livestock (MoFL)	Robiat Ferdausi,	Senior Assistant Secretary, MoFL
Ministry of Industries (MoInd)	Sanjay Kumar Ghosh	Deputy Secretary, MoInd
Department of Agricultural Extension (DAE)	Md. Obaidur Rahman Mondol	Director, Field service wing, DAE
Department of Agricultural Marketing (DAM)	Reza Ahmed Khan	Deputy Director, DAM
Bangladesh Standards and Testing Institute (BSTI)	Md. Saiful Islam	Director (Cm), BSTI
Health Services Division (HSD)	Sharaban Tahura	Joint Secretary, HSD
Bangladesh National Nutrition Council (BNNC)	Mr. Akther Imam,	Deputy Director (Monitoring & Evaluation), BNNC
Bangladesh Institute of Research and Training on Applied Nutrition (BIRTAN)	Mrs. Tasnima Mahjabin,	Senior Scientific Officer, BIRTAN
Ministry of Women and Children Affairs (MoWCA)	MOSAMMAT HASINA AKTER	Deputy Secretary Planning-2, Planning-3
General Economics Division (GED)	Mr. Mohammad Fahim Afsan Chowdhury,	Deputy Chief, (Poverty Analysis), GED
Ministry of Environment, Forest and Climate Change (MoEFCC)	Mohammad Razib Siddique	Deputy Secretary (CC-2), MoEFCC
Bangladesh Bureau of Statistics (BBS)	Md Alamgir Hossen	Deputy Director, BBS
Bangladesh Food Safety Authority (BFSA)	Professor Dr. Mohammad Shoeb	Member (Food Industry and Production), BFSA
Ministry of Chittagong Hill Tracts Affairs (MoCHTA)	Mohd. Monirul Islam	Additional secretary, MoCHTA
Economic Relations Division (ERD), Ministry of Finance	Bidhan Baral	Joint secretary, ERD
Cabinet division	Mohammad Khaled Hasan	Additional secretary, Cabinet division
Nethearland embassy	Osman Haruni	Senior Policy Advisor, Agriculture and Food Security, Trade and Business Development
International Centre for Climate Change and Development ICCCAD	Savio Rousseau Rozario	Researcher
International Centre for Climate Change and Development ICCCAD	Rawnak Jahan Khan Ranon	Researcher
International Centre for Climate Change and Development ICCCAD	Afshara Binte Mirza	
Sher-e-Bangla Agricultural University SAU	Dr. Mohammad Mizanul Haque Kazal	Professor
Sher-e-Bangla Agricultural University SAU	Md. Hayder Khan Sujan	Assistant Professor
Sher-e-Bangla Agricultural University SAU	Monira Sultana	
Bangladesh Agricultural University BAU	Dr. Mohammad Amirul Islam	Professor
Bangladesh Agricultural University BAU	Prof. Sadika Haque	Professor
Bangladesh Agricultural University BAU	Dr. Sheikh Mohammad Sayem	Associate Professor
Center for Environmental and Geographic Information Services CEGIS	Farhana Ahmed	Senior Specialist
Center for Environmental and Geographic Information Services CEGIS	Mohammad Abdur Rashid	Senior Specialist
Center for Environmental and Geographic Information Services CEGIS	Anindya Banik	Hydrologist
University of Dhaka	Dr. Nazma Shaheen	Professor
University of Dhaka	Saiful Islam	Associate Professor
University of Dhaka	Abira Nowar	Lecturer
